# Effectiveness of Therapeutic Exercise in Musculoskeletal Risk Factors Related to Swimmer’s Shoulder

**DOI:** 10.3390/ejihpe12060044

**Published:** 2022-06-02

**Authors:** Nuno Tavares, Gonçalo Dias, Paulo Carvalho, João Paulo Vilas-Boas, Maria António Castro

**Affiliations:** 1 Faculty of Sports, University of Porto and CIAFEL, 4200-450 Porto, Portugal; ftnunotavares@gmail.com; 2 Polytechnic Institute of Coimbra, Coimbra School of Education, Rua D. João III, Solum, 3030-329 Coimbra, Portugal; goncalodias@fcdef.uc.pt; 3 Research Unit for Sport and Physical Activity, University of Coimbra, 3004-531 Coimbra, Portugal; 4 Applied Research Unit (IIA), Polytechnic Institute of Coimbra, Robocorp, 3045-093 Coimbra, Portugal; 5 School of Health, Polytechnic of Porto, 4200-072 Porto, Portugal; paulocarvalho@ess.ipp.pt; 6 LABIOMEP-UP—Porto Biomechanics Laboratory, Faculty of Sports and CIFI2D, University of Porto, 4200-450 Porto, Portugal; jpvb@fade.up.pt; 7 School of Health Sciences, Polytechnic of Leiria, 2411-901 Leiria, Portugal; 8 CEMMPRE—Centre of Mechanical Engineering, Materials and Processes, University of Coimbra, 3030-788 Coimbra, Portugal

**Keywords:** swimming, therapeutic exercise, shoulder, swimmer’s shoulder, injury prevention

## Abstract

Background: Therapeutic exercise seems to minimize musculoskeletal risk factors related to swimmer’s shoulder. However, there is an absence of a qualitative evaluation of these programs and a great variability regarding the characteristics of exercises. The objective of this review was to identify, evaluate, and compare exercise programs used to minimize musculoskeletal risk factors related to swimmer’s shoulder. Methods: PubMed, CochraneLibrary, ScienceDirect, and Medscape were searched during January 2022. The research was limited to meta-analyses, systematic reviews, and RCTs written in English, Spanish, and Portuguese without publication time. The researched papers had swimmers as the population, compared an exercise program with another program or without intervention, and had as outcomes musculoskeletal risk factor variables. Results: Eleven articles were considered for analysis. There were three positive effects of strength programs on shoulder rotators’ strength and endurance, two positive effects of strength and stretching programs on shoulder posture, and one positive effect of a stretching program on shoulder ROM and a plyometric program on proprioception. Conclusions: There is high heterogeneity and little methodological quality evidence about the theme. However, strength programs with five or fewer OKC exercises performed out of the water seems to lead to better results in the swimmer’s shoulder prevention.

## 1. Introduction

Elite swimmers swim around 14,000 m daily, which results in about 2500 shoulder revolutions each workout and 16,000 each training week. This large amount of shoulder revolutions can easily overload soft tissue structures around the shoulder and create an overuse injury [1]. Swimmer’s shoulder is the most common injury in swimmers and is defined as a painful syndrome that occurs mostly in the anterior region of the shoulder and results from repetitive impingement of the rotator cuff under the coracoacromial arch during the technical gesture of swimming [1,2]. It is estimated that 91% of competitive swimmers experience this injury during their sports career [1,3], leading to the major cause of missed practice [2,3,4,5]. Described in the literature are several types of risk factors related to swimmer’s shoulder such as musculoskeletal, training, epidemiological, physiological, and lifestyle factors [1,2,3,5,6,7,8] (Table 1). Musculoskeletal risk factors have great importance in the clinical context because they are modifiable factors and some of them have good power to predict this injury [8].

In the last two decades, several studies have been carried out to test some prevention exercise programs to minimize swimmer’s shoulder musculoskeletal risk factors [9,10,11,12,13,14,15,16,17,18,19]. Most of them have had a positive effect favorable to therapeutic exercise when compared to no intervention, leading to the assertion that therapeutic exercise seems to be one of the principal tools available to prevent this injury [9,10,11,12,13,14]. Mostly, the impact of these therapeutic exercise programs has been studied on variables that characterize the strength and endurance of the shoulder musculature [9,11,12,13,15,16,17,18,19], which seems to be the musculoskeletal risk factor with the highest rate of injury prediction [8]. However, although in a minority, some studies have investigated the impact of therapeutic exercise in other important swimmer’s shoulder risk factors such as posture [10,11], proprioception [9], shoulder range of motion [14], and scapular dyskinesia [17]. The great variability regarding the characteristics of the prevention exercise programs tested, as well as the lack of a detailed qualitative evaluation of each study, makes it impossible to extract strong conclusions that guide and support the clinical practice. Currently, although therapeutic exercises are used in the swimming context, there are no criteria about the best type of exercise, what characteristics it should have, or what its purpose is in the context of prevention. This adds interest to the present study that attempted to fulfil the lack of information about the real effectiveness of therapeutic exercises for swimmers’ shoulder musculoskeletal risk factors.

The main objective of this systematic review was to identify, evaluate, and compare the effectiveness of different therapeutic exercise programs reported in the scientific literature in changing musculoskeletal risk factors related to swimmer’s shoulder: strength and endurance, shoulder posture, proprioception, glenohumeral instability, shoulder range of motion (ROM), and scapular dyskinesia.

## 2. Materials and Methods

The protocol followed in this review began with the search and selection of individual studies, quality assessment of the studies, data collection, analysis and interpretation of results, and extraction of conclusions. To structure the systematic review, the PRISMA recommendations were used [20] (Table S1). This systematic review was carried out with the participation of 5 researchers. Two researchers carried out the entire process of creating the systematic review and making the final decision on the selection of studies, and the others were responsible for supervising the process and the final evaluation of the review.

### 2.1. Eligibility Criteria

The research was limited to meta-analyses, systematic reviews, and randomized controlled trials (RCTs) written in English, Spanish, or Portuguese and published in scientific journals. There were no restrictions on the year of publication of the articles sought. The population considered for the searched articles included competitive swimmers of any age without shoulder pain. All articles with athletes from other water sports such as water polo, synchronized swimming, surf, or sports that combined swimming with other sports activities such as aquathlon or triathlon were excluded. Studies that performed a comparison between a therapeutic exercise program with another program or with no intervention were sought. The outcomes were variables that characterize musculoskeletal risk factors described in Table 1. All articles that did not meet the criteria presented above related to study design, language, population, intervention, comparison, and outcomes were excluded from this systematic review. All studies that investigated the acute effects of therapeutic exercise were excluded, and only articles that studied the effect of intervention applied for a minimum of 2 weeks were accepted.

### 2.2. Information Sources

To carry out the bibliographic research, PubMed, Cochrane Library, ScienceDirect, and Medscape databases were consulted in the period from January 1st to 31st in the year 2022.

### 2.3. Search Strategy

The criteria applied to each database, as well as the descriptors used in the research, are listed in Table 2.

### 2.4. Selection Process

After performing the bibliographic search, repeated articles were verified and excluded. Then, all titles were read and studies whose population were not swimmers or whose anatomical region under study was not the shoulder were removed. Subsequently, the abstracts were read and all articles that did not have the qualifying study design for this systematic review were excluded. Furthermore, all articles whose intervention was not a therapeutic exercise program and whose results were not variables that characterize musculoskeletal risk factors related to swimmer’s shoulder were also excluded. Finally, the articles were read in full, and the studies that only investigated the acute effect of the therapeutic exercise were removed.

### 2.5. Data Collection Process

The present data in this systematic review were collected by reading the selected articles, and it was used the information expressed in the published full text. Article identification, eligibility criteria, number of participants, sample characteristics, study design, type of intervention, the outcome of interest, number of dropouts, statistical significance and if available, study limitations were collected.

### 2.6. Data Items

The independent variables of this review were swimmers and therapeutic exercise programs. All variables characterizing musculoskeletal risk factors for swimmer’s shoulder were dependent variables:

- Strength and endurance: peak torque (PT) or peak force (PF), time to PT or PF, PT or PF to body weight ratio, torque decrement, amortization time, conventional and functional agonist–antagonist PT or PF ratios, and fatigue index.
- Shoulder posture: acromial distance, forward head angle, total scapular distance, and pectoralis minor length.
- Proprioception: joint position sense (JPS), kinesthesia, and force sense.
- Glenohumeral instability: inferior, anterior, and posterior displacement of the humeral head.
- Shoulder ROM: physiological shoulder ROM of internal rotation (IR), external rotation (ER), flexion (FLX), extension (EXT), adduction (ADD), abduction (ABD), horizontal adduction (HADD), and horizontal abduction (HABD); glenohumeral internal rotation deficit (GIRD), total rotational range of motion (TRROM), posterior shoulder tightness (PST), and humeral torsion (HT).
- Scapular dyskinesia: mean difference in the position of the scapula evaluated in: IR and ER, elevation and depression, protraction and retraction, anterior and posterior tipping, and upward and downward rotation.

### 2.7. Study Risk of Bias Assessment

The assessment of the risk of bias in the studies included in this review was performed by applying the PEDro scale criteria [21]. Whenever the article was already classified in the Physiotherapy Evidence Database (https://pedro.org.au accessed on 13 February 2022), this score was accepted. When this did not happen, the article was read in its entirety and subsequently evaluated by two authors (N.T. and M.A.C.), using the available criteria. The final score higher than 7 was attributed to a study with “high quality”, between 5 and 6 for “moderate quality” was considered, and scores lower than 4 were of “low quality” [22].

Additionally, the RoB2 tool (https://riskofbias.info/welcome/rob-2-0-tool accessed on 20 February 2022) from Cochrane was used. The “robvis (visualization tool)” (https://mcguinlu.shinyapps.io/robvis accessed on 20 February 2022) was used to access the risk of bias characterization charts of the individual studies. A generic evaluation grid was chosen and built two types of charts: a summary plot—where an assessment of certain biases was carried out in each study, and a traffic light plot—which revealed the global percentage of studies that contained the different biases.

### 2.8. Effect Measures

If possible, the measures collected from the individual studies were the effect size, mean difference between sample groups, the respective confidence interval, and its statistical significance (*p*-value).

### 2.9. Certainty Assessment

Certainty assessment of the positive effects was performed by two authors (N.T. and M.A.C.) based on four domains of GRADE assessment: risk of bias, inconsistency, indirectness, and imprecision [23].

## 3. Results

### 3.1. Study Selection

**Figure 1:**
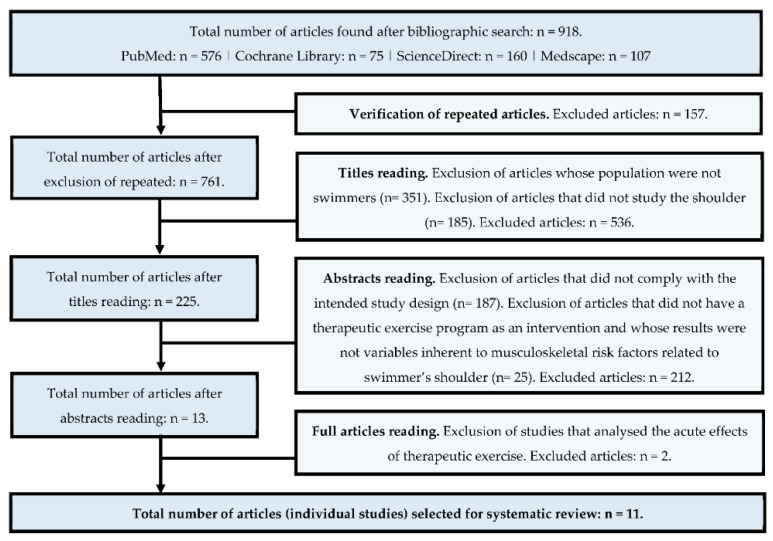
Study selection process for the systematic review.

After consulting PubMed, Cochrane Library, ScienceDirect, and Medscape databases, a total of 918 articles were found, of which 157 were excluded because they were repeated in more than one database. Then, the title was read and all articles that did not have swimmers as a population and that did not investigate the shoulder were excluded, leaving 225 articles. Of these publications, the abstract was read, and all study designs that were not meta-analyses, systematic reviews, and RCTs were excluded. In the same way, articles that did not compare an exercise program with another program or with no intervention and that did not investigate the impact of exercise on the variables that characterize the musculoskeletal risk factors in swimmer’s shoulder were also excluded. Finally, the 13 remaining articles were fully read, and 2 studies were excluded from this systematic review as they investigated the immediate effects of a therapeutic exercise intervention (Figure 1).

### 3.2. Study Characteristics

The characteristics of the individual studies are described in Table 3.

### 3.3. Risk of Bias in Studies

**Figure 2:**
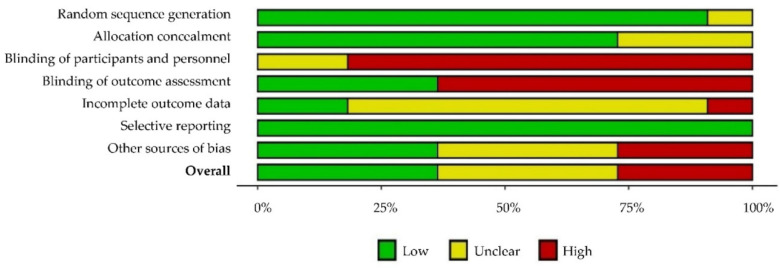

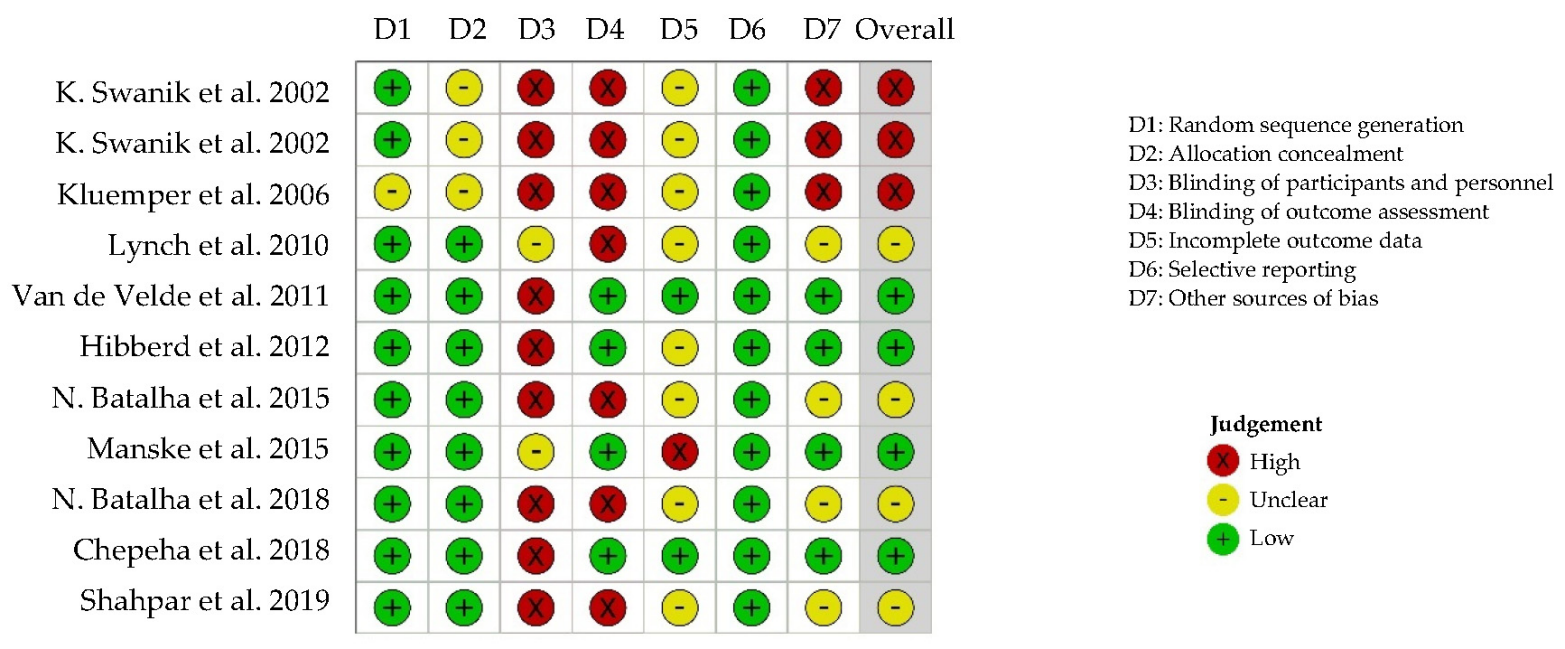
Risk of bias assessment using RoB2: generic—summary plot and traffic light plot [9,10,11,12,13,14,15,16,17,18,19].

The assessment of the risk of bias in the individual studies is described in Table 4 and Figure 2.

### 3.4. Results of Individual Studies

#### 3.4.1. Strength Program vs. No Intervention

After performing a 6 week program of plyometric exercises, significant differences were visible in the experimental group in the time to PT of the shoulder IR evaluated at 60°/s (*p* = 0.020) and 240°/s (*p* = 0.001), in the time of amortization evaluated at 450°/s (*p* = 0.008), and in torque decrement rated at 240°/s (*p* = 0.002). There were no differences between groups in the PT to body weight ratio of IR and ER/IR ratio [9]. In turn, the application of a strength program over 16 weeks led to significant mean differences verified in the experimental group in the PT of the ER: 2.93 ± 2.83 N.m, *p* = 0.008 (dominant side at 60°/s); 3.23 ± 1.68 N.m, *p* = 0.015 (non-dominant side at 60°/s); and 2.81 ± 2.66, *p* = 0.015 (dominant side at 180°/s), and in the ER/IR ratio: 10.31 ± 9.98, *p* = 0.001 (dominant side at 60°/s); 10.31 ± 9.98, *p* = 0.036 (non-dominant side at 60°/s); and 12.18 ± 8.66, *p* = 0.020 (dominant side at 180°/s). No significant differences were visible between groups in the PT of IR [12]. Similarly, the realization of a strength program for 12 weeks also showed a significant difference (*p* ≤ 0.05) found in the experimental group in the isometric maximum strength of the ER. After the intervention, the experimental group increased 1.19 ± 0.55 kg, which is equivalent to a 23% difference compared to the initial evaluation, and the control group increased 0.46 ± 0.63 kg; that is, an 11% difference compared to the initial evaluation [13]. In the opposite direction, the performance of a 6 week program of functional training did not lead to any significant difference between the experimental and control groups regarding the PT to body weight ratio, tested at 180°/s and 300°/s in IR, ER, HADD, HABD, and Serratus punch [15].

#### 3.4.2. Strength and Stretching Program vs. No Intervention

Although the application of the combined strength and stretching program over 6 weeks resulted in differences in maximal strength of the shoulder flexors (*p* = 0.020) and abductors (*p* = 0.014) in the intervention group, this was not considered statistically significant using the Bonferroni test. There were also significant gains in maximal strength in the shoulder extensors (*p* = 0.005) and muscles responsible for scapular retraction (*p* < 0.005), but this occurred similarly in both groups [17]. Similarly, another combined strength and stretching program did not cause significant differences between the control and experimental group in maximal strength of the middle trapezius, lower trapezius, and serratus anterior. When performing an analysis between the initial and final evaluation, there were visible improvements in the maximum strength of these muscles (*p* < 0.05); however, this increase occurred identically in both groups and may be related to the intensity of swimming training [11].

#### 3.4.3. Strength Program vs. Strength Program

The 12 week strength program compared with a 12 week endurance program caused significant improvements in maximal strength and scapular protraction–retraction ratio in both sample groups. However, these programs also led to a significant increase in the fatigue index for protraction on both sides (*p* = 0.05) and retraction on the non-dominant side (*p* = 0.009). Therefore, there seems to be a tendency for both programs to have positive effects on strength outcomes, but not on endurance outcomes [16]. In another study, the effectiveness of a dry-land strength program was compared with another strength program performed in water for 10 weeks. The final evaluation showed significant differences (*p* < 0.05) in the group that performed aquatic exercises in the PT of the IR in both shoulders tested at 60°/s and in the non-dominant shoulder tested at 180°/s. However, this intervention induced a significant decrease in the ER/IR ratios in this experimental group, leading to an imbalance in the shoulder stabilizing muscles of the swimmers. Additionally, the authors described a significant decrease in the ER fatigue ratio of both shoulders (*p* < 0.05) in the group that performed a dry-land exercise program [18]. Another research compared the effectiveness of an 8 week combined stretching and strength program in an open kinetic chain with another stretching and strength program in a closed kinetic chain and with a control group. The study concluded that open and closed kinetic chain exercises improved (*p* < 0.05) the PT of ER and IR significantly at all speeds tested—60°/s, 120°/s, and 180°/s. The improvement in strength was more pronounced in the group that performed the open kinematic chain exercise program [19].

#### 3.4.4. Shoulder Posture

After performing a 6 week strength and stretching program, a significant decrease of 9.6 ± 7.3 mm (*p* < 0.05) in the acromial distance measured in the standing relaxed position was observed in the experimental group [10]. Likewise, after applying an 8 week strength and stretching program, a significant reduction was observed in the anterior angle of the head (*p* = 0.005) and in the anterior translation of the shoulder (*p* = 0.001) in the experimental group. No differences were found between groups regarding scapular distance [11].

#### 3.4.5. Proprioception

The application of a plyometric program for 6 weeks led to significant improvements in the experimental group in the two components of proprioception evaluated. Regarding JPS, there was a significant improvement in the 0° ER during the ER movement (*p* = 0.015); 75° ER during the ER (*p* = 0.013) and IR (*p* = 0.007) movements; and 90% of the maximum ER position during the ER (*p* = 0.032) and IR (*p* = 0.003) movements. In the kinesthesia, significant differences were visible in all measurements evaluated: 0° ER to IR (*p* = 0.016) and ER (*p* = 0.003), 75° ER to IR (*p* = 0.028) and ER (*p* = 0.001), and 90% of the maximum ER to IR (*p* = 0.001) and ER (*p* = 0.003) [9].

#### 3.4.6. Shoulder ROM

Before and after 4 and 8 weeks of a sleeper stretch, IR and HADD ROM were evaluated in the shoulders. The results indicated a significant gain in the experimental group in dominant shoulder IR ROM (*p* < 0.001) and in GIRD (*p* < 0.001) after 4 and 8 weeks; and in non-dominant shoulder IR ROM (*p* = 0.03), dominant shoulder HADD ROM (*p* = 0.003), and non-dominant shoulder HADD ROM (*p* = 0.05) after 8 weeks [14].

#### 3.4.7. Shoulder Dyskinesia

After an execution of the combined strength and stretching program for 6 weeks, it was observed that the differences between the scapular kinematics variables were insignificant. Although there seems to be a tendency for the experimental group to achieve a greater scapular IR at 0° and 30° of flexion and some differences in scapular elevation and depression, this was not considered a significant interaction [17].

### 3.5. Certainty of Evidence

The effectiveness of a strength program on shoulder rotators’ strength and endurance [9,12,13] and strength and stretching program on shoulder posture [10,11] were classified as moderate evidence because they present a high risk of bias—an average of 4/10 and 3/10 on the PEDro scale. The other two positive effects were classified as low evidence. The first problem is related to the fact that it is impossible to classify the consistency in both cases because there was only one study that reported a positive effect. Additionally, both samples have limitations in their constitution. The effect of a plyometric program [9] was observed only in female swimmers and the effect of a stretching program [14] was studied in a small and poorly characterized sample. Finally, in the first case, there was also a high risk of bias—3/10 on the PEDro scale (Table 5).

## 4. Discussion

### Summary of Evidence

The main objective of this review was to identify, evaluate, and compare the different therapeutic exercise programs used to minimize musculoskeletal risk factors related to swimmer’s shoulder.

Eleven articles were considered for analysis. There were three positive effects of strength programs on shoulder rotators’ strength and endurance [9,12,13], two positive effects of strength and stretching programs on shoulder posture [10,11], and one positive effect of a stretching program on shoulder ROM [14] and a plyometric program on proprioception [9]. In general, it was observed that there is high heterogeneity in the genesis of these investigations. This fact led to the creation of a systematic review without meta-analyses. One of the examples of this huge variability is in maximal strength. This variable appeared in six different studies [11,12,13,16,18,19], and the authors used two different outcomes for their characterization (PT and PF), two different assessment instruments (isokinetic dynamometer and manual dynamometer), and three different units (Nm, N, and Kg). Additionally, when evaluating the sample groups of these six studies, it was observed that some have compared a therapeutic exercise program with a control group [11,12,13], and others with another therapeutic exercise program under different conditions [16,18,19].

Regarding the qualitative evaluation of the articles, 7 of the 11 studies [9,10,11,12,15,18,19] had low quality, which necessarily leads to the conclusions of this systematic review having to be viewed with some caution (Table 4). The blinding of the participants and the blinding of the evaluators were the main biases found in the individual studies, with a prevalence of high risk of bias above 75% and 50%, respectively (Figure 2).

Strength and endurance were the most analyzed risk factors in individual studies [9,11,12,13,15,16,17,18,19]. All strength programs that caused significant differences in favor of the experimental group had five or fewer exercises [9,12,13], and the only study where this was not observed had seven exercises [15]. When strength programs were tested alone compared to a control group, there seemed to be a positive effect in favor of the experimental group, especially in maximal ER strength [12,13]. In contrast, some studies have applied a combined strength and stretching program that did not show significant changes in strength and endurance variables [11,17]. Lastly, strength programs performed out of the water [18] and with open kinematic chain exercises [19] seems to lead to more significant improvements in strength and endurance outcomes.

In contrast, there were a small number of studies investigating the influence of therapeutic exercise on other musculoskeletal risk factors [9,10,11,14,17]. In two of these studies, the effect of a combined strength and stretching program on shoulder posture was observed, with significant differences favorable to the experimental group in the acromial distance [10], anterior shoulder translation, and in the head forward angle [11]. Although it lacks more robustness, there seems to be a tendency for a strength program to improve some of the components of proprioception [9] and a stretching program to contribute to a better balance in rotation ROM between the dominant and non-dominant shoulder [14]. The analysis of the influence of exercise in the improvement of scapular dyskinesia in swimmers did not show a significant difference; however, this risk factor was only evaluated after a very extensive intervention that combined strength and stretching exercises [17]. No studies were found that explore the impact of therapeutic exercise on glenohumeral instability in swimmers.

About the characteristics of exercise programs, due to their enormous variability, it is difficult to extract strong conclusions. Strength exercises were usually performed with elastic bands [9,10,12,13,15,17,18], focusing mostly on the ER, IR, middle trapezius, lower trapezius, and serratus anterior. All therapeutic exercise programs found had a minimum duration of 6 weeks [9,10,11,12,13,14,15,16,17,18,19], and normally were carried out 2–3 times a week [9,10,11,12,13,15,16,17,18,19]. The most frequently reported volume for strength exercises was three sets of 10 or 15 repetitions [9,10,11,15,16,19]. In turn, muscle stretching exercises tended to be static, with the pectoralis minor being the most targeted muscle [10,11,17,19] (Table 3). Five of seven positive effects considered in this review had progressions over time [9,10,12,13].

The main limitation of this systematic review is the high heterogeneity of the analyzed evidence, making it impossible to carry out a meta-analysis of the results, combined with the low methodological quality of the individual studies, which decreases the reliability of the conclusions obtained.

## 5. Conclusions

Therapeutic exercise is a strong and safe tool that can be used in the clinical practice of swimming to reduce the risk of injury associated with some musculoskeletal factors. Strength programs, with five or fewer exercises, performed out of the water and with OKC exercises, seem to lead to more improvements in the strength and endurance of the shoulder rotators, and possibly reduce the impact of this musculoskeletal risk factor on swimmer’s shoulder. This program should last for 6 weeks, be carried out 2–3 times a week, and have progressions over time. Programs that combine strength and stretching exercises seem to improve some variables that characterize shoulder posture, but not strength and endurance.

However, there is evidence with high heterogeneity and low methodological quality regarding the effectiveness of therapeutic exercise programs on musculoskeletal risk factors related to swimmer’s shoulder. In the future, more studies are needed to give better consistency and robustness to the conclusions obtained in this review regarding shoulder strength, endurance, and posture. Further investigations are essential to verify the impact of therapeutic exercise on other musculoskeletal risk factors related to swimmer’s shoulder, such as proprioception, shoulder ROM, scapular dyskinesia, and glenohumeral instability.

## Figures and Tables

**Table 1:** Summary table of main risk factors related to swimmer’s shoulder.

Type	Risk Factors
Musculoskeletal	Strength and endurance [2,5,7,8], shoulder posture [1,2,5,8], proprioception [1,2], glenohumeral instability [1,2,7], shoulder range of motion [1,2,5,8], scapular dyskinesia [1,2,8].
Training	Load [2], volume [2,3,8], intensity [2], style [2,5], distance [2,6], technique [7,8], years of experience [2], breathing side [2], training equipment [2], competitive level [2,8], cross training [2], stretching [2].
Epidemiological	Previous injuries [2,3], sex [2], age [2,3].
Psychological	Subjective pain perception and beliefs [3,8].
Lifestyle	Social status [2].

**Table 2:** Databases, criteria applied, and descriptors used in the bibliographic research.

Database	Applied Criteria	Descriptors
PubMed	#1 and #2 without MeSH terms and #3 with MeSH terms.	#1: (“swimming” OR “swimmer”) AND (“shoulder” OR “shoulder injury” OR “swimmer’s shoulder”)
		#2: (“swimming” OR “swimmer”) AND (“shoulder” OR “shoulder injury” OR “swimmer’s shoulder”) AND (“prevention” OR “injury prevention” OR “injury risk factors” OR “risk factor”)
		#3: (“swimming”) AND (“shoulder pain” OR “shoulder injuries”) AND (“exercise” OR “exercise therapy” OR “exercise movement techniques”) AND (“muscle strength” OR “range of motion, articular” OR “joint instability” OR “proprioception” OR “posture”)
Cochrane Library	Limited on study design: Cochrane reviews and trials.	#4: “swimming” AND “shoulder”
		#5: “swimming” AND “shoulder pain”
Science Direct	Limited on study design: review and research articles and in research area: medicine and dentistry and nurse and health professions.	#6: (“swimming”) AND (“shoulder pain”) AND (“exercise” OR “exercise therapy”) AND (“risk factor OR prevention”)
Medscape	Research carried out in the Medline section	#7: (“swimming” OR “swimmer”) AND (“shoulder” OR “shoulder pain” OR “swimmer’s shoulder”) AND(“exercise” OR “exercise therapy”)

**Table 3:** Individual study characteristics: population, intervention, and conclusion.

Study	Sample	Interventions	Name of Exercises	Exercise Program Characteristics	Conclusion
K. A. Swanik et al., 2002 [9]	24 competitive female swimmers with an average of 20.0 ± 1.1 years.	EG: Plyometric exercises. CG: No intervention.	Concentric IR using an elastic tube. Pitchback system: throw and catch a weighted ball.	Duration: 6 wks. Frequency: 2× wk. Sets: 3. Reps: 15. Progressions: 1st 2 wks—elastic band; after—pitchback system (ball weight 2–8 Kg), cycles—1 throwing every 2 s. Moment of realization: No reference. Monitoring: Investigator.	Significant improvement after the plyometric program in kinesthesia and some muscle performance characteristics.
K. A. Swanik et al., 2002 [15]	26 swimmers	EG: Functional training. CG: No intervention.	Elastic-tubing exercises: IR at 90°, ER at 90°, HABD and FLX, HADD and EXT. Prone exercises: ER with 120° ABD, ER with 90° ABD. Push-up plus.	Duration: 6 wks. Frequency: 3× wk. Sets: 3. Reps: 15. Progressions: started—2nd resistance level elastic band and 2 Kg prone exercises; progresses when performing all sets without difficulty. Moment of realization: Preseason. Monitoring: Swimming coach.	No differences were found between groups in muscle performance parameters. There was a significant decrease in the incidence of shoulder pain.
Kluemper et al., 2006 [10]	39 competitive swimmers with a mean age of 16 ± 2 years.	EG: Strength and stretching exercises. CG: No intervention	Strength exercises: Scapular retraction, ER at 90°, Ys. Stretching exercises: Pectoral minor stretching, pectoralis major stretching.	Duration: 6 wks. Frequency: 3× wk. Strength—Sets: 3. Reps: 15. Stretching—Sets: 2. Duration: 30 s. Progressions: Initial resistance—5 reps of each strength exercise; wk1—3 × 10; wk2—3 × 15; wk3—3 × 20; wk4—3 × 10 + difficult elastic band; wk5—3 × 15; wk6—3 × 20. Moment of realization: After warm-up. Monitoring: Investigators.	The EG significantly reduced the distance from the acromion to the wall in the relaxed standing position compared to the CG.
Lynch et al., 2010 [11]	28 competitive swimmers between 17 and 23 years	EG: Strength and stretching exercises. CG: No intervention	Strength exercises: Y to W, L to Y, elbow push-up. Stretching exercises: Pectoralis flexibility, chin tucks.	Duration: 8 wks. Frequency: 3× wk. Strength—Sets: 3. Reps: 10. Stretching—Sets: 10. Duration: 5 s. Progressions: No progressions. Moment of realization: During regular dry-land training. Monitoring: Investigator.	The intervention carried out led to a decrease in head and shoulder forward posture in young competitive swimmers.
Van de Velde et al., 2011 [16]	18 swimmers with an average of 14.7 ± 1.3 years.	G1: Strength exercises. G2: Endurance exercises.	Serratus anterior: Dynamic hug movement, elbow push-up. Lower trapezius: ER with a dumbbell, bilateral HABD + scapular retraction with 2 dumbbells.	Duration: 12 wks. Frequency: 3× wk. Strength—Sets: 3. Reps: 10. Endurance—Sets: 3. Reps: 20. Progressions: Loads assessment after 6 wks. Moment of realization: Before water training. Monitoring: Physiotherapist.	Both exercises programs increased absolute strength, but neither had a positive effect on scapular muscle endurance parameters.
Hibberd et al., 2012 [17]	37 young competitive swimmers.	EG: Strength and stretching exercises. CG: No intervention	Strength: Shoulder FLX, Shoulder EXT, IR at 90°, ER at 90°, throwing acceleration, throwing deceleration, low scapular rows, scapular punches, Ys, Ts, Ws. Stretching: Sleeper stretch, corner stretch.	Duration: 6 wks. Frequency: 3× wk. Strength—Sets: 2. Reps: 15. Stretching—Sets: 2. Duration: 30 s. Progressions: No progressions. Moment of realization: After swimming training. Monitoring: Swimming coach.	There were no significant changes in the strength of the glenohumeral and scapular musculature and scapular kinematics between the groups.
N. Batalha et al., 2015 [12]	40 competitive male swimmers aged between 14 and 15 years.	G1: Strength exercises. G2: No exercise. GC: Sedentary youth.	Exercises with TheraBand: Ws, Ys, shoulder press.	Duration: 16 wks. Frequency: 3× wk. Sets: 3, 30 s rest. Reps: 20—1st 2 sets, maximum possible—3rd set. Progressions: TheraBand resistance was increased after 30 reps in the 3rd set. Moment of realization: During training. Monitoring: No reference.	Significant increases in strength of shoulder IR and an improvement in the balance of the rotators, especially at 60°/s speed.
Manske et al., 2015 [13]	43 young swimmers under 18 years.	EG: Strength exercises. CG: No intervention	Exercises with TheraBand: Shoulder ABD, shoulder EXT, shoulder IR, shoulder ER.	Duration: 12 wks. Frequency: 2/3× wk. Sets: 2. Reps: 15.Progressions: Initial TheraBand resistance assessment— progressively test of all resistances, athletes referring 0–10 difficulty of execution, ideal resistance when felt > 6; next progression when the athlete felt < 6 after exercises. Moment of realization: Before water training. Monitoring: Investigators.	Swimmers belonging to the EG had a significantly increased strength of shoulder ER compared to the CG.
N. Batalha et al., 2018 [18]	25 young swimmers aged between 12 and 15 years.	G1: Dry-land strength exercises. G2: In-water strength exercises.	Dry-land exercises: Ws, Ys, ER at 90°. In-water exercisers: ER with Theraband, ER with hand paddles, sculling.	Duration: 10 wks. Frequency: 3× wk. Dry-land—Sets: 3, 30 s rest. Reps: 20—1st 2 sets, maximum possible—3rd set. Progressions: TheraBand resistance was increased after 30 reps in the 3rd set. In-water—Sets: 3–5, 10 s rest. Progressions: Every 2 wks—wk1—3 × 30 s; wk3—4 × 30 s; wk5—3 × 45 s; wk7—4 × 45 s; wk9—5 × 30 s. Moment of realization: No reference. Monitoring: Swimming coach.	A dry-land strength program was found to be more effective in improving shoulder rotator balance and ER endurance compared to an in-water program.
Chepeha et al., 2018 [14]	8 swimmers aged between 18 and 35 years.	EG: Stretching exercise. CG: No intervention	Sleeper stretch.	Duration: 8 wks. Frequency: 7× wk. Sets: 5, 1–2 min rest. Duration: 2 min. Progressions: No progressions. Moment of realization: No reference. Monitoring: Swimming coach— swimmer’s performance, physiotherapist—assessment.	There was an increase in dominant shoulder IR and HADD ROM.
Shahpar et al., 2019 [19]	45 competitive male swimmers aged between 18 and 25 years.	G1: Stretching and strength exercises in OKC. G2: Stretching and strength exercises in CKC. CG: No intervention.	Warm-up: Run or cycle. Pendular arm movements, posterior deltoid stretching, passive IR, passive ER, sleeper stretch, corner stretch. Strength in OKC: ER, IR, dumbbell fly, reverse dumbbell fly. Strength in CKC: Push up, scapular push up, scapular dip, crab walk.	Duration: 8 wks. Frequency: 3× wk. Stretching—Sets: 1–3. Reps: 4–10, 30 s rest. Strength in OKC—Sets: 3. Reps: 8–15, 3 min rest. Strength in CKC—Sets: 3. Reps: 6–8, 1–3 min rest. Progressions: Performed regularly over time, strength training load 80–90% of 1RM. Moment of realization: No reference. Monitoring: No reference.	Both exercise programsincreased the strength of shoulder ER and IR in swimmers. The authors also suggested that OKC exercises are more effective compared to CKC exercises.

EG—Experimental group|CG—Control group|G1—Group 1|G2—Group 2|wk—week|wks—weeks|Reps—Repetitions|s—seconds|IR—Internal rotation|ER—External rotation|FLX—Flexion|EXT—Extension|ABD—Abduction|HABD—Horizontal abduction|HADD—Horizontal adduction|ROM—Range of motion|OKC—Open kinetic chain|CKC—Closed kinetic chain.

**Table 4:** Risk of bias assessment of individual studies using the PEDro scale.

	Risk of Bias Assessment Criteria—PEDro Scale Item												
	1 *	2	3	4	5	6	7	8	9	10	11	Final Score	Quality
K. Swanik et al., 2002 [9]	•	•								•	•	3/10	Low
K. Swanik et al., 2002 [15]	•	•								•	•	3/10	Low
Kluemper et al., 2006 [10]	•									•	•	2/10	Low
** Lynch et al., 2010 [11]	•	•								•	•	4/10	Low
Van de Velde et al., 2011 [16]	•	•		•				•		•	•	5/10	Moderate
** Hibberd et al., 2012 [17]	•	•		•			•			•	•	5/10	Moderate
N. Batalha et al., 2015 [12]	•	•		•						•	•	4/10	Low
** Manske et al., 2015 [13]	•	•		•			•			•	•	5/10	Moderate
N. Batalha et al., 2018 [18]	•	•		•						•	•	4/10	Low
** Chepeha et al., 2018 [14]	•	•		•			•	•		•	•	6/10	Moderate
Shahpar et al., 2019 [19]	•	•		•						•	•	4/10	Low
**PEDro Scale Item** 1. Eligibility criteria specified 2. Random allocation 3. Concealed allocation 4. Group similar at baseline 5. Subject blinding 6. Therapist blinding 7. Assessor blinding 8. Less than 15% dropouts 9. Intention-to-treat analysis 10. Between-group statistical comparisons 11. Point measures and variability data													

* PEDro scale criteria n°1 does not contribute to the final score. ** The final score was obtained through the Physiotherapy Evidence Database (PEDro).

**Table 5:** Certainty of evidence assessment.

Positive Effects	Risk of Bias	Inconsistency	Indirectness	Imprecision	Certainty of Evidence
Strength program on shoulder rotators’ strength/endurance	Downgraded	Not downgraded	Not downgraded	Not downgraded	Moderate
Strength and stretching programs on shoulder posture	Downgraded	Not downgraded	Not downgraded	Not downgraded	Moderate
Plyometric program on proprioception	Downgraded	-	Downgraded	Not downgraded	Low
Stretching program on shoulder ROM	Not downgraded	-	Downgraded	Not downgraded	Low

## Data Availability

Data sharing not applicable.

## Supplementary Materials

The following supporting information can be downloaded at: https://www.mdpi.com/article/10.3390/ejihpe12060044/s1, Table S1: PRISMA 2020 Checklist.
